# Novel gel-flush rescue technique for an impacted detachable snare sheath in the working channel during polypectomy

**DOI:** 10.1055/a-2888-9970

**Published:** 2026-06-23

**Authors:** Koichi Hamada, Masafumi Ishikawa, Yoshinori Horikawa, Kae Techigawara, Takayuki Nagahashi, Takuro Nakaya, Akane Yamabe

**Affiliations:** 1 Department of Gastroenterology 13704 Southern Tohoku Research Institute for Neuroscience, Southern Tohoku General HospitalKoriyamaFukushimaJapan; 2 Department of Minimally Invasive Surgical and Medical Oncology 12775 Fukushima Medical UniversityFukushimaFukushimaJapan

Detachable snares prevent bleeding during endoscopic resection of pedunculated polyps 1​ and achieving hemostasis in colonic diverticular bleeding. 2​ Mechanical failures (e.g., inability to release the loop) rarely occur. 3​ Typically, the device is cut proximally, the endoscope is withdrawn, and the loop is severed upon reinsertion. However, if the cut sheath completely retracts into the working channel and becomes jammed, endoscope withdrawal applies direct traction to the polyp stalk, creating a high risk of unintended transection, bleeding, or perforation.

A 54-year-old woman underwent polypectomy for an approximately 20-mm pedunculated polyp in the ascending colon ( Fig. 1 ) ( Video 1 ). A detachable snare (PolyLoop Ligating Device; Olympus, Tokyo, Japan) applied to the stalk prevented bleeding ( Fig. 2a ). As the loop could not be released ( Fig. 2b ), the device was cut proximally ( Fig. 3 ). Immediately after the sheath was retracted into the channel, strong resistance was felt upon attempting scope withdrawal. To prevent perforation from excessive tension on the polyp, withdrawal was aborted. Strong resistance inside the instrument port prevented pushing the sheath with grasping forceps. Flushing water (1 mPa·s) through the port was ineffective because of leakage around the sheath. We forcefully injected a highly viscous gel (VISCOCLEAR; Otsuka Pharmaceutical Factory, Tokushima, Japan) directly into the biopsy valve using a 50-mL syringe. The gel’s high viscosity (768 mPa·s) 4​ physically sealed the gaps, effectively transmitting the hydraulic pressure from the syringe into a forward propulsive force, successfully extruding the jammed sheath into the colon. After safe scope withdrawal and reinsertion, the polyp stalk was resected between the ligated loop and mucosal base using a scissor-type knife. The polyp was retrieved along with the snare without complications ( Fig. 4 ). This novel technique, utilizing the gel’s high viscosity and hydraulic pressure, is a simple and highly effective rescue strategy for jammed devices.

**Fig. 1:**
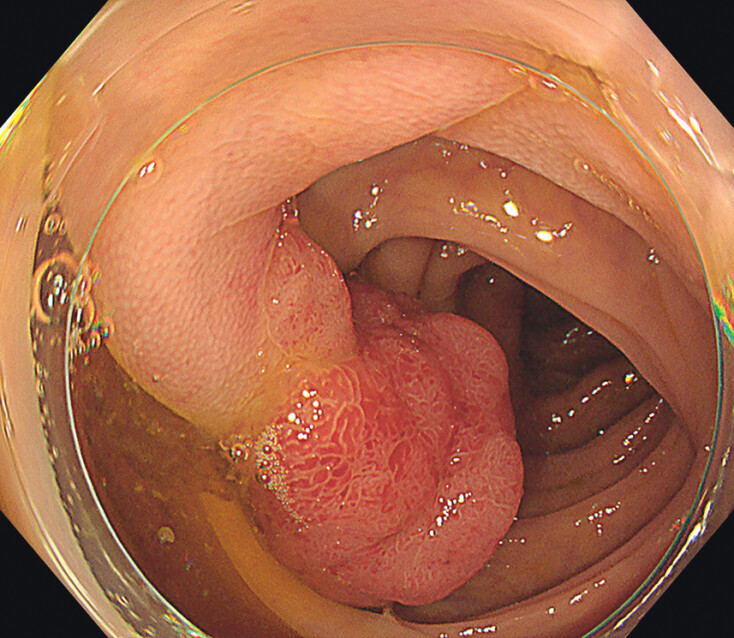
White light image showing a pedunculated polyp, approximately 20 mm in size, in the ascending colon.

**Video 1** Novel gel-flush rescue technique for an impacted detachable snare sheath in the working channel during polypectomy.


**Fig. 2:**
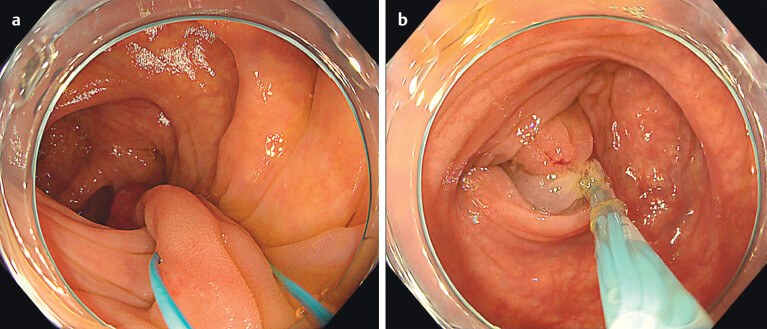
( **a** ) Polyp stalk ligation using a detachable snare. ( **b** ) Failure of the loop release, showing the wire abnormally coiled within the sheath.

**Fig. 3:**
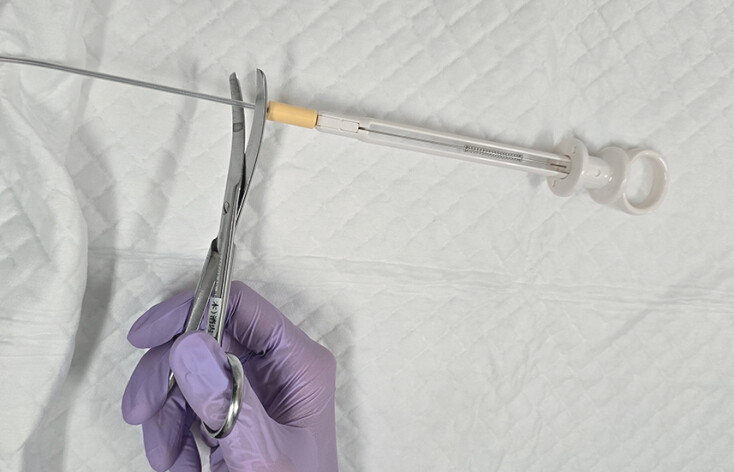
Extracorporeal transection of the device (benchtop demonstration). The sheath of the detachable snare was cut with scissors near the handle.

**Fig. 4:**
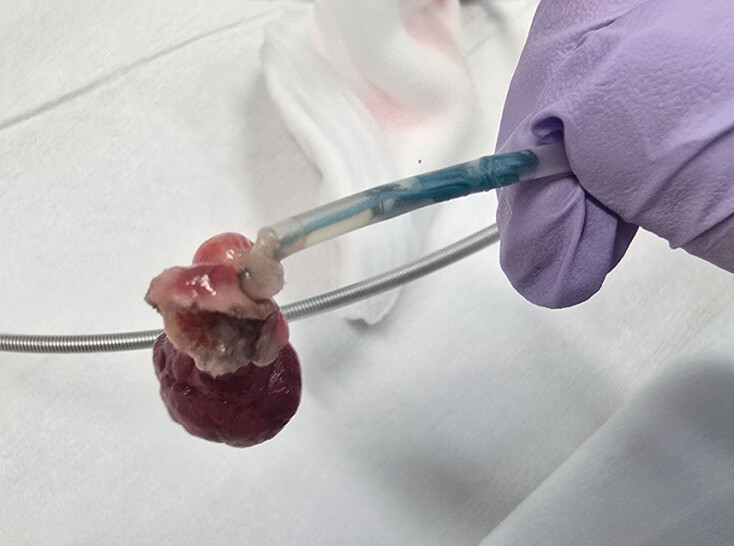
Macroscopic view of the retrieved specimen. The detachable snare was tightly ligated to the polyp and the wire was abnormally coiled within the sheath.

Endoscopy_UCTN_Code_TTT_1AQ_2AD_3AB

## References

[1] Iishi H Tatsuta M Narahara H Endoscopic resection of large pedunculated colorectal polyps using a detachable snare Gastrointest Endosc 1996 44 594 597 8934168 10.1016/s0016-5107(96)70015-9

[2] Hamada K Kawano K Nishida S Endoscopic detachable snare ligation therapy for colonic diverticular hemorrhage improves procedure time compared to endoscopic band ligation Turk J Gastroenterol 2022 33 443 448 35678803 10.5152/tjg.2022.21494 PMC11157994

[3] Silva R A Dias L M Brandão C A new technique for releasing a stuck loop Endoscopy 2006 38 437 16680659 10.1055/s-2006-925100

[4] Nakazawa K Hirata Y Koshiba R Removal of jammed loop cutter and residual snare wire, after polypectomy with a detachable snare Endoscopy 2024 56 E959 E960 39515770 10.1055/a-2439-3733 PMC11548996

